# Complete genome sequence of *Planctomyces brasiliensis* type strain (DSM 5305^T^), phylogenomic analysis and reclassification of *Planctomycetes* including the descriptions of *Gimesia* gen. nov., *Planctopirus* gen. nov. and *Rubinisphaera* gen. nov. and emended descriptions of the order *Planctomycetales* and the family *Planctomycetaceae*

**DOI:** 10.1186/1944-3277-9-10

**Published:** 2014-12-08

**Authors:** Carmen Scheuner, Brian J Tindall, Megan Lu, Matt Nolan, Alla Lapidus, Jan-Fang Cheng, Lynne Goodwin, Sam Pitluck, Marcel Huntemann, Konstantinos Liolios, Ioanna Pagani, Konstantinos Mavromatis, Natalia Ivanova, Amrita Pati, Amy Chen, Krishna Palaniappan, Cynthia D Jeffries, Loren Hauser, Miriam Land, Romano Mwirichia, Manfred Rohde, Birte Abt, John C Detter, Tanja Woyke, Jonathan A Eisen, Victor Markowitz, Philip Hugenholtz, Markus Göker, Nikos C Kyrpides, Hans-Peter Klenk

**Affiliations:** 1 DSMZ - German Collection of Microorganisms and Cell Cultures GmbH, Braunschweig, Germany; 2 DOE Joint Genome Institute, Walnut Creek, California, USA; 3 Los Alamos National Laboratory, Bioscience Division, Los Alamos, New Mexico, USA; 4 Biological Data Management and Technology Center, Lawrence Berkeley National Laboratory, Berkeley, California, USA; 5 Oak Ridge National Laboratory, Oak Ridge, Tennessee, USA; 6 Jomo Kenyatta University of Agriculture and Technology, Juja, Kenya; 7 HZI – Helmholtz Centre for Infection Research, Braunschweig, Germany; 8 University of California Davis Genome Center, Davis, California, USA; 9 Australian Centre for Ecogenomics, School of Chemistry and Molecular Biosciences, The University of Queensland, Brisbane, Australia; 10 Department of Biological Sciences, King Abdulaziz University, Jeddah, Saudi Arabia

**Keywords:** Non-peptidoglycan bacteria, Stalked bacteria, Halotolerant, Gram-negative, Taxonomic descriptions, *Planctomycetales*, *Planctomycetes*, GEBA

## Abstract

*Planctomyces brasiliensis* Schlesner 1990 belongs to the order *Planctomycetales*, which differs from other bacterial taxa by several distinctive features such as internal cell compartmentalization, multiplication by forming buds directly from the spherical, ovoid or pear-shaped mother cell and a cell wall consisting of a proteinaceous layer rather than a peptidoglycan layer. The first strains of *P. brasiliensis*, including the type strain IFAM 1448^T^, were isolated from a water sample of Lagoa Vermelha, a salt pit near Rio de Janeiro, Brasil. This is the second completed genome sequence of a type strain of the genus *Planctomyces* to be published and the sixth type strain genome sequence from the family *Planctomycetaceae*. The 6,006,602 bp long genome with its 4,811 protein-coding and 54 RNA genes is a part of the ***G*** *enomic* ***E*** *ncyclopedia of* ***Bacteria*** *and* ***Archaea*** project. Phylogenomic analyses indicate that the classification within the *Planctomycetaceae* is partially in conflict with its evolutionary history, as the positioning of *Schlesneria* renders the genus *Planctomyces* paraphyletic. A re-analysis of published fatty-acid measurements also does not support the current arrangement of the two genera. A quantitative comparison of phylogenetic and phenotypic aspects indicates that the three *Planctomyces* species with type strains available in public culture collections should be placed in separate genera. Thus the genera *Gimesia*, *Planctopirus* and *Rubinisphaera* are proposed to accommodate *P. maris*, *P. limnophilus* and *P. brasiliensis*, respectively. Pronounced differences between the reported G + C content of *Gemmata obscuriglobus*, *Singulisphaera acidiphila* and *Zavarzinella formosa* and G + C content calculated from their genome sequences call for emendation of their species descriptions. In addition to other features, the range of G + C values reported for the genera within the *Planctomycetaceae* indicates that the descriptions of the family and the order should be emended.

## Introduction

Strain IFAM 1448^T^ (=DSM 5305 = ATCC 49424 = JCM 21570) is the type strain of *Planctomyces brasiliensis*. Although the genus currently consists of six species with validly published names, only three of them, *P. brasiliensis*, *P. limnophilus*, and *P. maris* contain cultured type strains [1-3]. The other three species, including the type species *P. bekefii*[4] as well as *P. stranskae* and *P. guttaeformis*[5] are to date not represented by cultured strains and have been described solely on the basis of their morphological properties [6], with the descriptions and illustrations serving as the type material. *P. bekefii* was initially described as a fungus under the International Code of Botanical Nomenclature [4,7]. The genus name derives from the Greek words ‘planktos’, wandering, floating, and ‘mukês’ meaning ‘fungus’ to indicate a floating fungus [4], reflecting their initial descriptions as members of the fungi. The species epithet ‘brasiliensis’ is a Latin masculine adjective that means “of or belonging to Brazil” [1]. Strain IFAM 1448^T^ together with other strains designated IFAM 1450, IFAM 1454 and IFAM 1456 were isolated from water samples collected in November 1982 from Lagoa Vermelha, a salt pit at the Atlantic coast north of Rio de Janeiro, Brazil [1]. Another strain (DSM 11908**)** potentially belonging to *P. brasiliensis* was isolated from postlarvae of the Giant Tiger Prawn, *Penaeus monodon*, infected with the monodon baculovirus [8].

The similarity of genes of both cultured and uncultured *Planctomyces* species has been studied not only based on 16S rRNA gene sequences, but also using the RNase P RNA genes [9]. The unique compartmentalized cell structure shared by all *Planctomycetes* investigated so far is remarkable [10], as is the relatively large size of *Planctomycetes* genomes, which could be attributed to their free-living lifestyles [6]. The membrane organization of organisms belonging to the *Planctomycetes*, *Verrucomicrobia* and *Chlamydiae* superphylum is currently the subject of intense discussion in the literature, e.g. as an exception to [11] or as a variation of [12] the classical Gram-negative cell plan. *P. limnophilus* has been established as model organism for the phylum, as it is the first and only species of *Planctomycetes* that has been genetically modified [13,14]. Here we present a summary classification and a set of features for *P. brasiliensis* IFAM 1448^T^, together with the description of the complete genomic sequencing and annotation, as well as a re-assessment of the taxonomy of the group based on phylogenomic as well as traditionally sampled characters.

## Organism information

### Classification and features

#### 16S rRNA gene analysis

A representative genomic 16S rRNA gene sequence of strain DSM 5305^T^ was compared with the Greengenes database for determining the weighted relative frequencies of taxa and (truncated) keywords as previously described [15]. The most frequently occurring genus was *Planctomyces* (100.0%) (18 hits in total). Regarding the two hits to sequences from members of the species, the average identity within high-scoring segment pairs (HSPs) was 100.0%, whereas the average coverage by HSPs was 97.2%. Regarding the five hits to sequences from other members of the genus, the average identity within HSPs was 90.1%, whereas the average coverage by HSPs was 58.5%. Among all other species, the one yielding the highest score was *P. maris*, which corresponded to an identity of 90.3% and an HSP coverage of 63.5%. The highest-scoring environmental sequence was DQ015774 (‘Antarctic lake water clone ELB25-062’) [16], which showed an identity of 96.2% and an HSP coverage of 98.3%. The most frequently occurring keywords within the labels of environmental samples that yielded hits were ‘treatment’ (2.7%), ‘microbi’ (2.1%), ‘sediment’ (1.8%), ‘gut’ (1.8%) and ‘microbiom’ (1.7%) (232 hits in total). Environmental samples which yielded hits of a higher score than the highest scoring species were not found, which might indicate that *P. brasiliensis* is rarely found in the environment. But overall *Planctomyces* species have been isolated from an ecologically large variety of environments [2,3], which is also reflected by the above most frequently occurring keywords.

**Figure 1:**
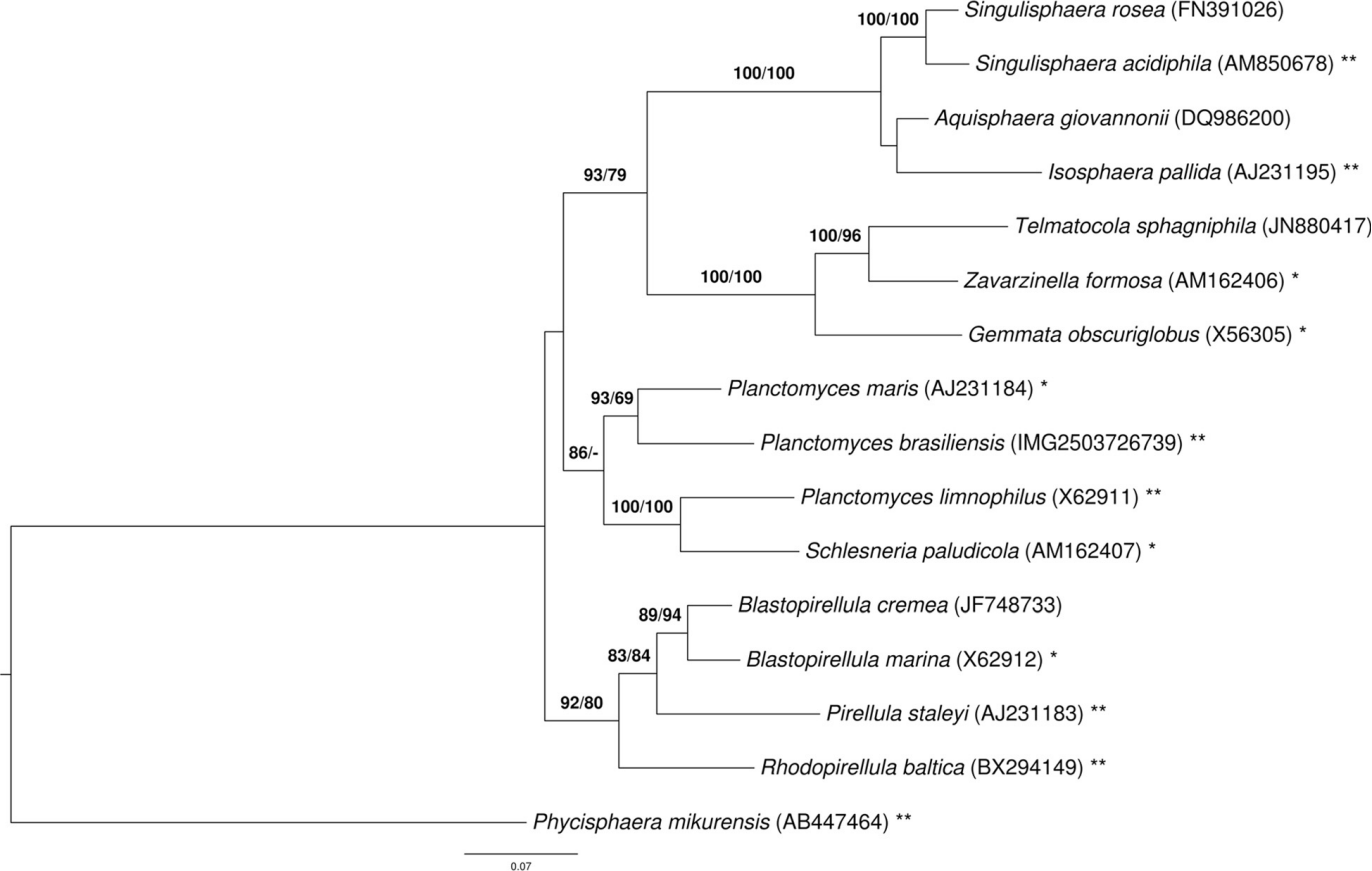
**Phylogenetic tree highlighting the position of *P. brasiliensis *relative to the other species within the family *Planctomycetaceae *.** The tree was inferred from 1,343 aligned characters of the 16S rRNA gene sequence under the maximum likelihood (ML) criterion as previously described [15]. Rooting was done initially using the midpoint method [17] and then checked for its agreement with the current classification (Table 1). The branches are scaled in terms of the expected number of substitutions per site. Numbers above the branches are support values from 400 ML bootstrap replicates (left) and from 1,000 Maximum-Parsimony bootstrap replicates (right) if larger than 60% [15]. Lineages with type strain genome sequencing projects registered in GOLD [18] as unpublished are marked with one star, those listed as published (as well as the target organism) with two stars [19-22].

Figure 1 shows the phylogenetic neighborhood of *P. brasiliensis* DSM 5305^T^ in a 16S rDNA based tree. The sequences of the two identical 16S rRNA gene copies in the genome do not differ from the previously published 16S rDNA sequence (AJ231190).

#### Morphology and physiology

**Figure 2:**
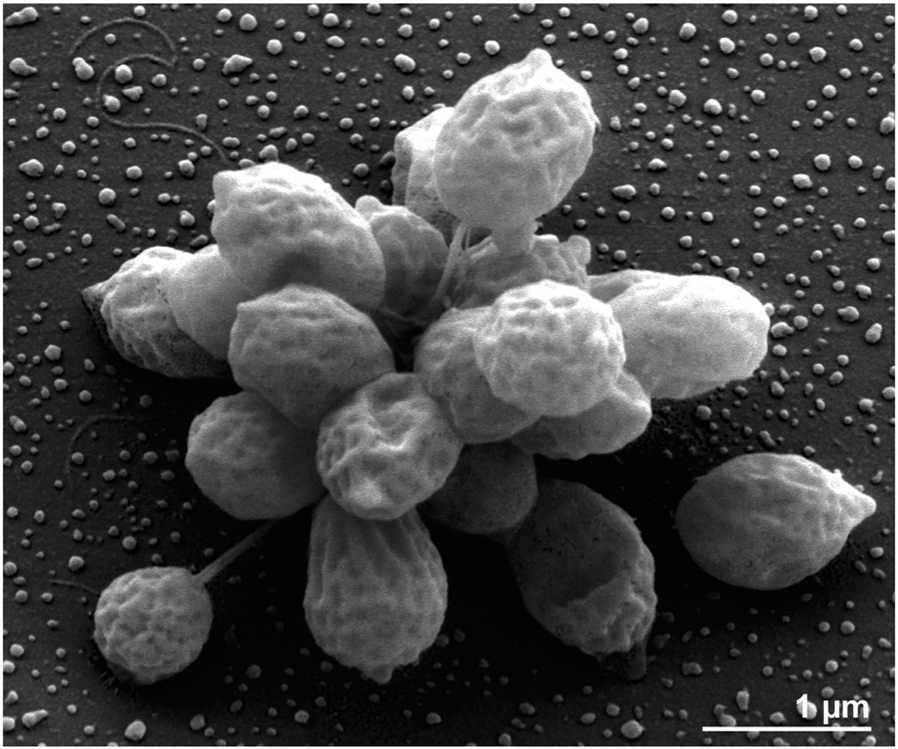
**Scanning-electron micrograph of *P. brasiliensis *** **DSM 5305**^**T **^**highlighting stalks and crateriform structures on the cell surface.**

The morphology and life cycle of the organism resembles those of *P. maris* and *P. limnophilus* rather than *P. guttaeformis* or *P. stranskae*[1]. Adult cells have an excreted stalk consisting of many loosely twisted fibrils at the distal end where the holdfast is located, enabling the cells to attach to surfaces or to attach to one another, aggregating to rosettes (Figure 2) [1]. The reproductive pole is located opposite the site of the stalk. At the free cell pole, the bud has a polar to sub-polar, thick flagellum with a diameter of about 20 nm. Both the mother cell and bud have crateriform structures scattered over the whole of the cell surface [1]. The cells are spherical to ovoid with a diameter of 0.7 to 1.8 μm, hence, the cells are larger than those of *P. limnophilus* or *P. maris*[1]. The colonies have a dry, rough surface and a yellow to ochre pigmentation [1]. *P. brasiliensis* requires NaCl for growth and has a broad tolerance to salt (0.1 to 1.7 mol Na^+^/l) [1]. The strain grows chemoorganotrophically (Table 1) on the following carbon sources: D-cellobiose, D(+)-glucose, D(+)-galactose, maltose, D(+)-mannose, melibiose, rhamnose, ribose, trehalose, N-acetyl glucosamine and glucuronate. Carbon sources not utilized are D(−)-fructose, D-fucose, D(−)-lyxose, α-D-melezitose, raffinose, L(−)-sorbose, D(+)-xylose, methanol, ethanol, glycerol, D(−)-mannitol, D(−)-sorbitol, acetate, fumarate, lactate, malate, pyruvate and succinate [1]. Strain IFAM 1448^T^ can utilize ammonia, nitrate and N-acetyl glucosamine as a nitrogen source and can hydrolyze gelatin and tween 80. It does not utilize L-glutamic acid, L-glutamine, L-serine amygdalin, gluconate or creatinine and does not require vitamins for growth. The organism is able to reduce nitrate to nitrite under anaerobic conditions, a trait that is also predicted from the genome sequence (phenotype prediction by the IMG Pathway browser) [1].

**Table 1:** **Classification and general features of *P. brasiliensis *** **DSM 5305**^**T **^**in accordance with the MIGS recommendations**[23]** as published by the Genome Standards Consortium **[24]

**MIGS ID**	**Property**	**Term**	**Evidence code**
	Current classification	Domain *Bacteria*	TAS [25]
		Phylum *Planctomycetes*	TAS [26]
		Class "*Planctomycetacia*"	TAS [26]
		Order "*Planctomycetales*"	TAS [27-29]
		Family *Planctomycetaceae*	TAS [27,29]
		Genus *Planctomyces*	TAS [4,28,30,31]
		Species *Planctomyces brasiliensis*	TAS [32]
		Type strain DSM 5305	TAS [1]
	Gram stain	negative	TAS [1]
	Cell shape	sphere shaped	TAS [1]
	Motility	motile	TAS [1]
	Sporulation	none	TAS [1]
	Temperature range	mesophile	TAS [1]
	Optimum temperature	27°C-35°C	TAS [1]
	Salinity	halotolerant	TAS [1]
MIGS-22	Oxygen requirement	aerobe	TAS [1]
	Carbon source	several sugars, such as D-cellobiose, maltose, trehalose	TAS [1]
	Energy metabolism	chemoorganotroph	TAS [1]
MIGS-6	Habitat	marine fresh water	TAS [1]
MIGS-15	Biotic relationship	free-living	NAS
MIGS-14	Pathogenicity	not reported	NAS
	Biosafety level	1	NAS [33]
	Isolation	water from salt pit	TAS [1]
MIGS-4	Geographic location	Lagoa Vermelha, Brazil	TAS [1]
MIGS-5	Sample collection time	November 1982	TAS [1]
MIGS-4.1	Latitude	−22.929	NAS
MIGS-4.2	Longitude	−42.390	NAS
MIGS-4.3	Depth	surface water	TAS [1]
MIGS-4.4	Altitude	0 m, sea level	NAS

Evidence codes - TAS: Traceable Author Statement (i.e., a direct report exists in the literature); NAS: Non-traceable Author Statement (i.e., not directly observed for the living, isolated sample, but based on a generally accepted property for the species, or anecdotal evidence). These evidence codes are from the Gene Ontology project [34].

### Chemotaxonomy

Chemical analysis of the cell wall of *P. brasiliensis* showed that proteins are the principal constituents with a value of 79.9% of the dry weight [35]. Detailed analysis of the protein composition indicated that the major amino acids were asparagine, threonine, serine, glutamine, proline, glycine and alanine [36]. Total extractable lipids represent 10% of the cell dry weight of strain IFAM 1448^T^[23]. The major fatty acids (>1%) are C_16:0_ (30.7%), C_16:1_ (25%), C_18:1_ (13.1%), C_20:1_ (9.9%), C_15:0_ (8.4%), C_18:0_ (5.6%), C_14:0_ (4.0%), C_17:0_ (1.3%) and C_17:1_ (1.8%) [36]. A comparison with other representatives of the *Planctomycetes* is given below.

## Genome sequencing and annotation

### Genome project history

This organism was selected for sequencing on the basis of its phylogenetic position [37,38], and is part of the ***G*** *enomic* ***E*** *ncyclopedia of* ***Bacteria*** *and* ***Archaea*** project [39]. The genome project is deposited in the Genome On Line Database [18] and the complete genome sequence is deposited in GenBank. Sequencing, finishing and annotation were performed by the DOE Joint Genome Institute (JGI) using state-of-the-art sequencing technology [40]. A summary of the project information is shown in Table 2.

**Table 2:** Genome sequencing project information

**MIGS ID**	**Property**	**Term**
MIGS-31	Finishing quality	Finished
MIGS-28	Libraries used	Three genomic libraries: one 454 pyrosequence standard library, one 454 PE library (14 kb insert size), one Illumina library
MIGS-29	Sequencing platforms	Illumina GAii, 454 GS FLX Titanium
MIGS-31.2	Sequencing coverage	124.8 × Illumina; 91.0 × pyrosequence
MIGS-30	Assemblers	Newbler version 2.0.00.20-PostRelease-10-28-2008-g-3.4.6, phrap
MIGS-32	Gene calling method	Prodigal 1.4, GenePRIMP
	INSDC ID	CP002546
	Genbank Date of Release	March 2, 2011
	GOLD ID	Gc01674
	NCBI project ID	47863
	Database: IMG-GEBA	2503707005
MIGS-13	Source material identifier	DSM 5305
	Project relevance	Tree of Life, GEBA

### Growth conditions and DNA isolation

A culture of DSM 5305^T^ was grown in DSMZ medium 607 (M13 *Verrucomicrobium* medium) [41] at 30°C. DNA was isolated from 0.5-1 g of cell paste using Jetflex Genomic DNA Purification Kit (GENOMED 600100) following the standard protocol as recommended by the manufacturer: Cell lysis was enhanced by adding 20 μl proteinase K for two hours at 58°C. DNA is available through the DNA Bank Network [42].

### Genome sequencing and assembly

The genome was sequenced using a combination of Illumina and 454 sequencing platforms. All general aspects of library construction and sequencing can be found at the JGI website [43]. Pyrosequencing reads were assembled using the Newbler assembler (Roche). The initial Newbler assembly consisting of 53 contigs in one scaffold was converted into a Phrap assembly (http://www.phrap.com) making fake reads from the consensus, to collect the read pairs in the 454 paired-end library. Illumina GAii sequencing data (3,029.9 Mb) were assembled with Velvet [44] and the consensus sequences were shredded into 1.5 kb overlapped fake reads and assembled together with the 454 data. 454 draft assembly was based on 164.2 Mb 454 draft data and all of the 454 paired end data. Newbler parameters were -consed -a 50 -l 350 -g -m -ml 20. The Phred/Phrap/Consed software package (http://www.phrap.com/) was used for sequence assembly and quality assessment in the subsequent finishing process. After the shotgun stage, reads were assembled with parallel phrap (High Performance Software, LLC). Possible mis-assemblies were corrected with gapResolution (http://www.jgi.doe.gov/), Dupfinisher, or sequencing cloned bridging PCR fragments with subcloning or transposon bombing (Epicentre Biotechnologies, Madison, WI) [45]. Gaps between contigs were closed by editing in Consed, by PCR and by Bubble PCR primer walks (J.-F. Chang, unpublished). A total of 156 additional reactions were necessary to close gaps and to raise the quality of the finished sequence. Illumina reads were also used to correct potential base errors and increase consensus quality using the Polisher software developed at JGI [46]. The error rate of the completed genome sequence is less than one in 100,000. Together, the combination of the Illumina and 454 sequencing platforms provided 285.0 × coverage of the genome. The final assembly contained 281,884 pyrosequence and 39,867,623 Illumina reads.

### Genome annotation

Genes were identified using Prodigal [47] as part of the DOE-JGI [48] genome annotation pipeline, followed by a round of manual curation using the JGI GenePRIMP pipeline [49]. The predicted CDSs were translated and used to search the National Center for Biotechnology Information (NCBI) nonredundant database, UniProt, TIGR-Fam, Pfam, PRIAM, KEGG, COG, and InterPro databases. Additional gene prediction analysis and functional annotation was performed within the Integrated Microbial Genomes - Expert Review (IMG-ER) platform [50].

### Genome properties

**Figure 3:**
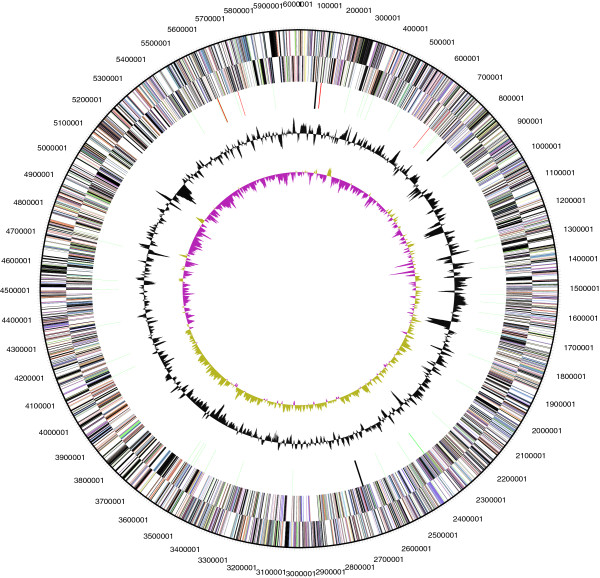
**Graphical circular map of chromosome.** From outside to the center: Genes on forward strand (colored by COG categories), Genes on reverse strand (colored by COG categories), RNA genes (tRNAs green, rRNAs red, other RNAs black), GC content, GC skew (purple/olive).

The genome consists of a 6,006,602 bp long chromosome with a G + C content of 56.5% (Table 3 and Figure 3). Of the 4,865 genes predicted, 4,811 were protein-coding genes, and 54 RNAs; 61 pseudogenes were also identified. The majority of the protein-coding genes (57.6%) were assigned with a putative function while the remaining ones were annotated as hypothetical proteins. The distribution of genes into COGs functional categories is presented in Table 4.

**Table 3:** Genome statistics

**Attribute**	**Value**	**% of total**
Genome size (bp)	6,006,602	100.00%
DNA coding region (bp)	5,145,779	85.67%
DNA G + C content (bp)	3,390,645	56.45%
Number of replicons	1	
Extrachromosomal elements	0	
Total genes	4,865	100.00%
RNA genes	54	1.11%
rRNA operons	2	
Protein-coding genes	4,811	98.89%
Pseudo genes	61	1.25%
Genes with function prediction	2,800	57.55%
Genes in paralog clusters	2,351	48.32%
Genes assigned to COGs	3,220	66.19%
Genes assigned Pfam domains	3,439	70.69%
Genes with signal peptides	645	13.26%
Genes with transmembrane helices	1,210	24.87%
CRISPR repeats	2	

**Table 4:** Number of genes associated with the general COG functional categories

**Code**	**Value**	**% age**	**Description**
J	149	4.0	Translation, ribosomal structure and biogenesis
A	3	0.0	RNA processing and modification
K	216	5.7	Transcription
L	223	5.9	Replication, recombination and repair
B	4	0.1	Chromatin structure and dynamics
D	32	0.9	Cell cycle control, cell division, chromosome partitioning
Y	0	0.0	Nuclear structure
V	83	2.2	Defense mechanisms
T	213	5.7	Signal transduction mechanisms
M	237	6.3	Cell wall/membrane/envelope biogenesis
N	193	5.1	Cell motility
Z	0	0.0	Cytoskeleton
W	0	0.0	Extracellular structures
U	250	6.7	Intracellular trafficking, secretion, and vesicular transport
O	140	3.7	Posttranslational modification, protein turnover, chaperones
C	166	4.4	Energy production and conversion
G	190	5.1	Carbohydrate transport and metabolism
E	208	5.6	Amino acid transport and metabolism
F	61	1.6	Nucleotide transport and metabolism
H	131	3.5	Coenzyme transport and metabolism
I	98	2.6	Lipid transport and metabolism
P	258	6.9	Inorganic ion transport and metabolism
Q	68	1.8	Secondary metabolites biosynthesis, transport and catabolism
R	461	12.3	General function prediction only
S	356	9.5	Function unknown
-	1,645	33.8	Not in COGs

## Insights from the genome sequence

### Taxonomic classification vs. 16S rRNA gene analysis of *Planctomyces* and *Schlesneria*

Although it has only slowly been appreciated by taxonomists after Darwin had published his seminal works, the sole possible goal of a taxonomic classification is to summarize the genealogy of the organisms [37,51]. For this reason, it does not matter if a taxonomic classification contains less information than the empirical estimate of the phylogeny from which it was derived. But it does matter whether or not a classification can pretend to summarize the respective underlying genealogies – and this is never the case where classifications include obviously non-monophyletic groups [37,51-53].

As indicated earlier [19], in this respect the current classification of *Planctomycetes* is only partially satisfactory because with the description of the genus *Schlesneria* the genus *Planctomyces* now appears to be paraphyletic in the 16S rRNA gene trees (see Figure 1 and [19-21,54]), with *P. limnophilus* being more closely related to *Schlesneria* than to the other *Planctomyces* species. The two versions of the 16S rRNA gene sequence of *Schlesneria paludicola* MPL7^T^ submitted to INSDC, NR_042466 and AM162407, are identical; AM162407 has been used in Figure 1. The previously published 16S rRNA gene sequences of *P. limnophilus*[19] and *P. brasiliensis* (this study) have also been found to be identical or almost identical to those from the respective genome-sequencing projects. For this reason, the biological identity of the *Schlesneria* and *Planctomyces* strains used can hardly be called into question, and a mix-up or contamination of cultures cannot explain the positioning of the strains in the phylogenetic analyses. In fact, that the establishment of the genus *Schlesneria* renders *Planctomyces* paraphyletic was, surprisingly, already visible in the phylogenetic tree presented in [55], even though support for the non-monophyly of *Planctomyces* is stronger in our analyses (Figure 1). (Since Kulichevskaya *et al.*[55] did not mention the issue of taxa being monophyletic this also cannot be considered to be a pre-requisite for their interpretation, even though it is then not entirely clear why they conducted a phylogenetic analysis in the first place.) This picture did not change after R + Y coding of the 16S rRNA gene alignment [56], which still yielded 97% support for the sister group of *P. limnophilus* and *S. paludicola* (data not shown).

To measure this phylogenetic conflict in detail, we conducted both unconstrained heuristic searches for the best tree under the maximum likelihood (ML) [57] and maximum parsimony (MP) criterion [58] as well as searches constrained for the monophyly of all genera (for details of the data matrix see the caption of Figure 1). The best-known ML tree had a log likelihood of −9,214.93, whereas the best tree found under the constraint had a log likelihood of −9,249.16. The constrained tree was significantly worse than the globally best one in the Shimodaira-Hasegawa test as implemented in RAxML [57] (α = 0.01). The best-known MP tree had a score of 1,519, whereas the best constrained trees found had a score of 1,541 and were significantly worse in the Kishino-Hasegawa test as implemented in PAUP* [58] (α = 0.05). (See, e.g. chapter 21 in [59] for an in-depth description of such paired-site tests.) Accordingly, the current classification within the family as used in, e.g., [54] is in significant conflict with the 16S rRNA gene data.

In the following we will assess whether these 16S rRNA gene results can be confirmed with genomic data.

### Phylogenomic analysis and comparative genomics

For the phylogenomic analysis, protein sequences from all available *Planctomycetaceae* and outgroup (*Phycisphaera mikurensis*) genomes were retrieved from NCBI or IMG (Table 5).

**Table 5:** **Genomic G + C content of the *Planctomycetaceae *** **type strains, including *Phycisphaera mikurensis *** **as outgroup**

	**G + C content from species description [%]**	**G + C content calculated from genome sequence [%]**	**NCBI accessions/IMG taxon IDs (number of contigs)**
*Blastopirellula marina* DSM 3645^T^	53.6 - 57.4 [60]	57.04*	AANZ00000000 (67); NZ_AANZ00000000 (4)*
*Gemmata obscuriglobus* UQM 2246^T^	64.4 ± 1.0 [61]	67.18	ABGO00000000 (923)
*Isosphaera pallida* ATCC 43644^T^	62.2 [62]	62.49 [20]	CP002353, CP002354
*Phycisphaera mikurensis* NBRC 102666^T^	73.0 [63]	73.22	AP012338, AP012339
*Pirellula staleyi* DSM 6068^T^	56.4 ± 0.4 - 57.4 ± 0.3 [64]	57.46 [21]	CP001848
*Planctomyces brasiliensis* DSM 5305^T^	55.1 - 57.7 [1]	56.45	CP002546
*Planctomyces limnophilus* DSM 3776^T^	53.24 ± 0.59 [2]	53.68 [19]	CP001744, CP001745
*Planctomyces maris* DSM 8797^T^	50.5 [65]	50.45	ABCE00000000 (126)
*Rhodopirellula baltica* SH 1^T^	55.0 [66]	55.40 [67]	BX119912
*Schlesneria paludicola* DSM 18645^T^	56.3 [55]	55.66*	AHZR00000000 (112); NZ_AHZR00000000 (24)*
*Singulisphaera acidiphila* DSM 18658^T^	59.9 [67]	62.23*	AGRX00000000 (113); CP003364 - CP003367*
*Zavarzinella formosa* DSM 19928^T^	62.5 [68]	59.10*	IMG ID: 2548877000 (594); NZ_AIAB00000000 (106)*

*Denote G + C content values calculated from the updated version of the genome sequence, which is also marked with an asterisk.

The G + C content calculated from the genome sequence of *G. obscuriglobus* was 67.18%, whereas the previously published value, determined using traditional techniques, is 64.4 ± 1.0% [61]. Similarly, the G + C content of *S. acidiphila* was given as 59.9% [66] and that of *Z. formosa* was given as 62.5% [68], whereas the analysis of the genome sequences yields 62.23% and 59.10%, respectively. The three strongly deviating G + C values discovered here were all obtained using thermal denaturation [61,67,68]. A recent study [69] has shown that when calculated from genome sequences the G + C content varies at most 1% within species (which holds even though no attempt was made to remove plasmid sequences) and that larger variances are caused by the inaccuracies of the traditional techniques. It has thus been recommended to conduct emendations of species descriptions in the case of discrepancies larger than 1%, and to also conduct emendations of genus descriptions if the species emendations yield values that do not fit into the range of the G + C content given in the literature for the respective genus [69].

The G + C content of the *Planctomycetaceae*, using exact calculations from the genome sequences where possible, ranges from 50% to 70% (Table 5 and [70,71]), similar to the range between 52% and 69% measured by Schlesner *et al*. using traditional methods [66]. However, the description of the order *Planctomycetales* as well as the family *Planctomycetaceae* by Schlesner and Stackebrandt 1986 [27], who could at that time only consider the *Pirellula staleyi*, *Pirellula marina* (formerly *Pirella*), *Planctomyces maris* and *Planctomyces limnophilus*, refers to a G + C content of 50 to 59%. *Planctomyces* and *Schlesneria* have a comparatively low G + C content between 50% and 56%, with *P. brasiliensis* and *P. maris* differing more strongly from each other than *P. limnophilus* and *S. paludicola*. Similarly, Schlesner *et al.* reported a G + C content of 50 to 58% for *Planctomyces* strains [66]. The G + C content of the group comprising *Blastopirellula*, *Pirellula* and *Rhodopirellula* ranges from 55% to 57%. Although the G + C content of *Zavarzinella* measured from the genome sequence is lower than the one given in the species description, the group containing *Aquisphaera*, *Gemmata, Isosphaera*, *Singulisphaera*, *Telmatocola* and *Zavarzinella* possesses a higher G + C content than the other *Planctomycetaceae*, between 59% and 70%.

The genome sequences (Table 5) were phylogenetically investigated using the DSMZ phylogenomics pipeline as previously described [72-75] using NCBI BLAST [76], TribeMCL [77], OrthoMCL [78], MUSCLE [79], RASCAL [80], GBLOCKS [81] and MARE [82] to generate gene- and ortholog-content matrices as well as concatenated alignments of distinct selections of genes. Maximum likelihood (ML) [83] and maximum-parsimony (MP) [84,85] trees were inferred from the data matrices with RAxML [57,86] and PAUP* [58], respectively, as previously described [72-75].

**Figure 4:**
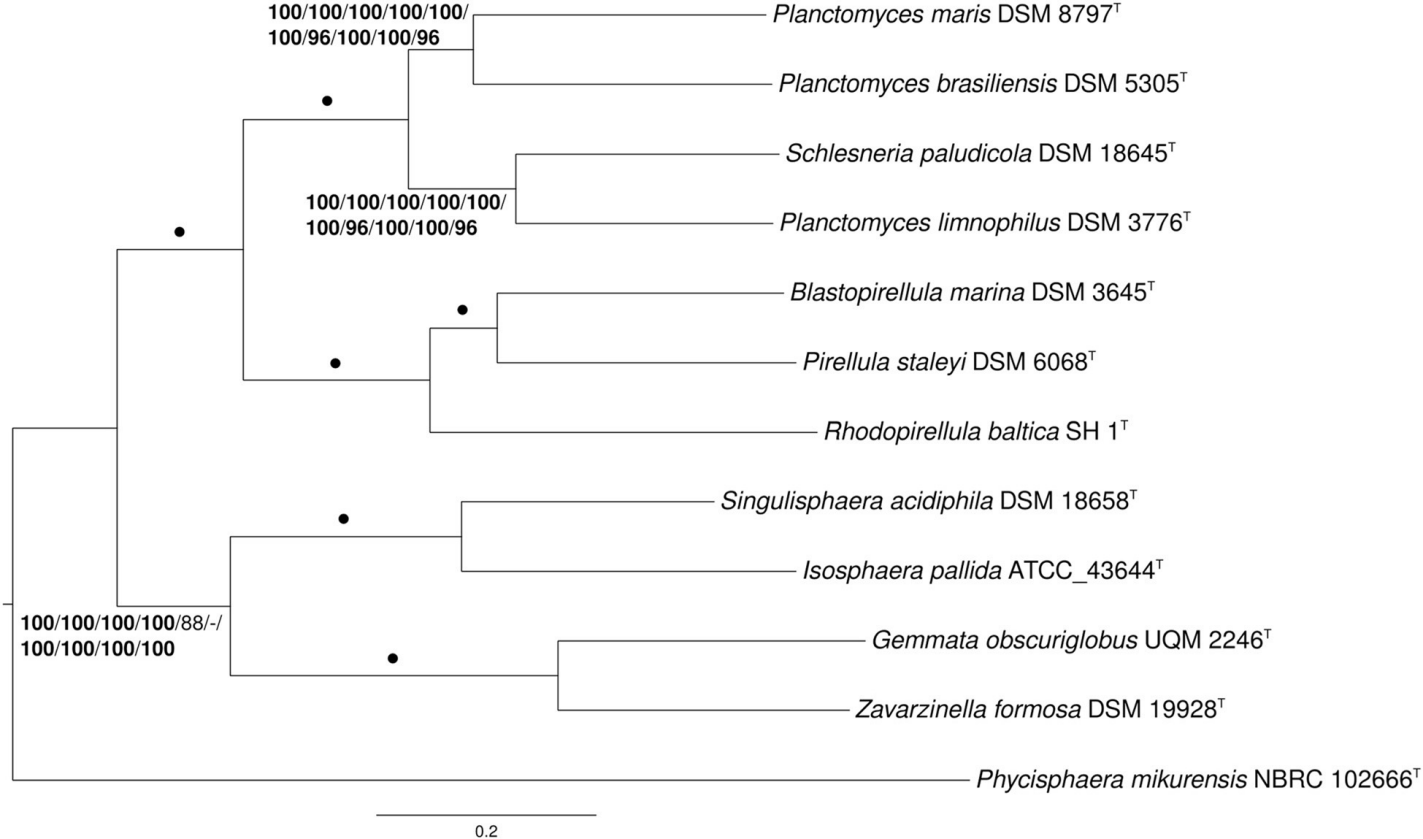
**Phylogenetic tree inferred from the MARE-filtered supermatrix under the maximum likelihood (ML) criterion **[82]** and rooted with *Phycisphaera mikurensis *.** The branches are scaled in terms of the expected number of substitutions per site. Numbers above the branches (from left to right) are bootstrapping support values [87] (if larger than 60%) from ML/MP MARE-filtered supermatrix; ML/MP unfiltered (full) supermatrix; ML/MP core-genes supermatrix; ML/MP gene-content matrix; ML/MP ortholog-content matrix. Values larger than 95% are shown in bold; dots indicate branches with maximum support under all settings.

The MARE-filtered supermatrix ML tree is shown in Figure 4 together with ML and MP bootstrap support values from all phylogenomic analyses if larger than 60%. All trees, except for the core-genes supermatrix MP tree, were topologically identical. The MP tree inferred from the core-gene matrix showed a distinct grouping of the clade that contained *Gemmata obscuriglobus* and *Zavarzinella formosa*, i.e. as sister clade of all other taxa but the outgroup, with maximal bootstrap support. Given that the majority of analyses supports the topology in Figure 4, the different core-genes MP topology might be caused by long branch attraction between the outgroup and the clade comprising *Gemmata* and *Zavarzinella*.

The phylogenomic tree (Figure 4) is topologically identical to the 16S rRNA gene tree (Figure 1) except for the backbone of the trees, which showed no support in the 16S rRNA gene analysis. Furthermore, the phylogenomic analysis confirms with maximum support the 16S rRNA tree that *P. limnophilus* and *S. paludicola* are sister taxa and, thus, *Planctomyces* is paraphyletic. The genomic interrelationships between these four species are thus examined more closely in the following. A genome sequence-based phylogeny with the same branching order but less bootstrapping support (probably because a restricted set of genes was used) was recently reported by Gou *et al*. [88].

The genomes of the four genome-sequenced *Planctomyces* and *Schlesneria* species differ significantly in their size. The genomes of *S. paludicola* (8.6 Mb, 6,860 protein-coding genes) and *P. maris* (7.8 Mb, 6,480 protein-coding genes) are significantly larger in size than the genomes of *P. brasiliensis* (6.0 Mb, 4,811 protein-coding genes) and *P. limnophilus* (5.5 Mb, 4,304 protein-coding genes).

To estimate the overall similarity between the four *Planctomycetaceae* genomes the GGDC (Genome-to-Genome Distance Calculator) [89,90] was used. The system calculates the distances by comparing the genomes to obtain HSPs (high-scoring segment pairs) and interfering distances from a set of formulas (1, HSP length / total length; 2, identities / HSP length; 3, identities / total length). Table 6 shows the results of the pairwise comparison of the *Planctomycetaceae* species with formula 2, as this formula is robust against the use of draft genomes such as ABCE00000000 (*P. maris*) [91].

**Table 6:** **Pairwise comparison of the four *Planctomycetaceae *** species using GGDC, formula 2 (DDH estimates based on identities / HSP length)*

	***P. brasiliensis***	***P. limnophilus***	***P. maris***	***S. paludicola***
*P. brasiliensis*	100.00%	18.60% ± 2.27	21.40% ± 2.34	20.90% ± 2.33
*P. limnophilus*	18.60% ± 2.27	100.00%	24.10% ± 2.39	23.20% ± 2.38
*P. maris*	21.40% ± 2.34	24.10% ± 2.39	100.00%	22.00% ± 2.35
*S. paludicola*	20.90% ± 2.33	23.20% ± 2.38	22.00% ± 2.35	100.00%

*The confidence intervals indicate the inherent uncertainty in estimating DDH values from intergenomic distances based on models derived from empirical test data sets (which are always limited in size); see [91] for details. The distance formulas are explained in [89]; formula 2 is recommended, particularly for draft genomes.

This comparison of the genomes revealed that *P. brasiliensis* shows a slightly higher DDH estimate with *P. maris*, compared to those with *P. limnophilus* and *Schlesneria paludicola*. For *Schlesneria paludicola*, a higher DDH value was estimated with *P. limnophilus*, in contrast to the other genomes. These results are in accordance with the 16S rRNA (Figure 1) and phylogenomic analyses (Figure 4). However, given the confidence intervals (Table 6), the DDH estimates do not show significant differences.

**Figure 5:**
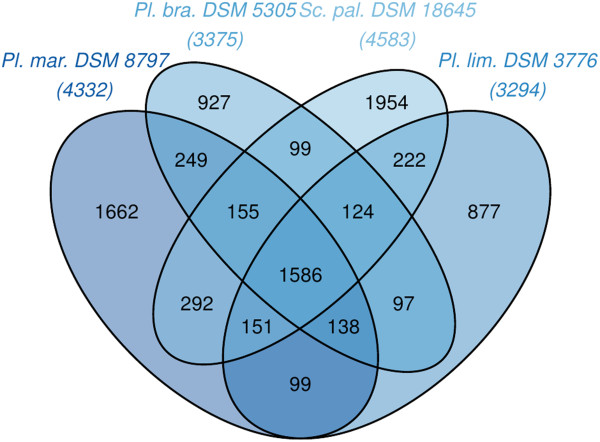
**Venn diagram depicting the intersections of sets of homologous proteins of *P. maris*, *P. brasiliensis*, *P. limnophilus *and *S. paludicola*.** Their cardinalities are given in parentheses; for the total number of proteins see Table 3 and the resources listed in Table 5. The Venn diagram was calculated with the corresponding R package [92].

The fraction of shared genes in the four genomes is shown in a Venn diagram (Figure 5). The numbers of pairwise shared genes were inferred from the TribeMCL analysis via counting the clusters of homologous genes shared by the genomes. 1,586 clusters of homologs are shared by all four *Planctomycetaceae* species.

*P. brasiliensis* shares 63.1% of its clusters with *P. maris,* but only 57.6% and 58.2% clusters with *P. limnophilus* and *S. paludicola*, respectively. Only 27.5% of the *P. brasiliensis* genes have no homologs in the other genomes. *P. limnophilus* shares 63.2% of its clusters with *S. paludicola*, whereas *P. brasiliensis* shares 58.2% and *P. maris* shares 50.4% clusters with *S. paludicola*.

*P. brasiliensis* shares many more clusters of homologous genes with *P. maris* than with the other genomes. Of the four compared genomes, *P. limnophilus* shares most clusters of homologous genes with *S. paludicola*. These results are in accordance with the 16S rRNA (Figure 1) and phylogenomic analyses (Figure 4) as well as the GGDC results (Table 6).

Jeske *et al*. [93] performed a comprehensive genome mining approach to identify secondary metabolite related genes or gene clusters within the *Planctomycetes*. Just as in the other studied *Planctomycetes*, a number of genes putatively related with the production of secondary metabolites were identified in *P. brasiliensis*, that is, bacteriocin, ectoine, lantipeptide, terpene and type 1 polyketide synthases [93]. The study revealed *Planctomycetes* as a rich source for small molecules that might ultimately lead to new antibiotics or drugs [93].

### Phenotypic comparison

**Figure 6:**
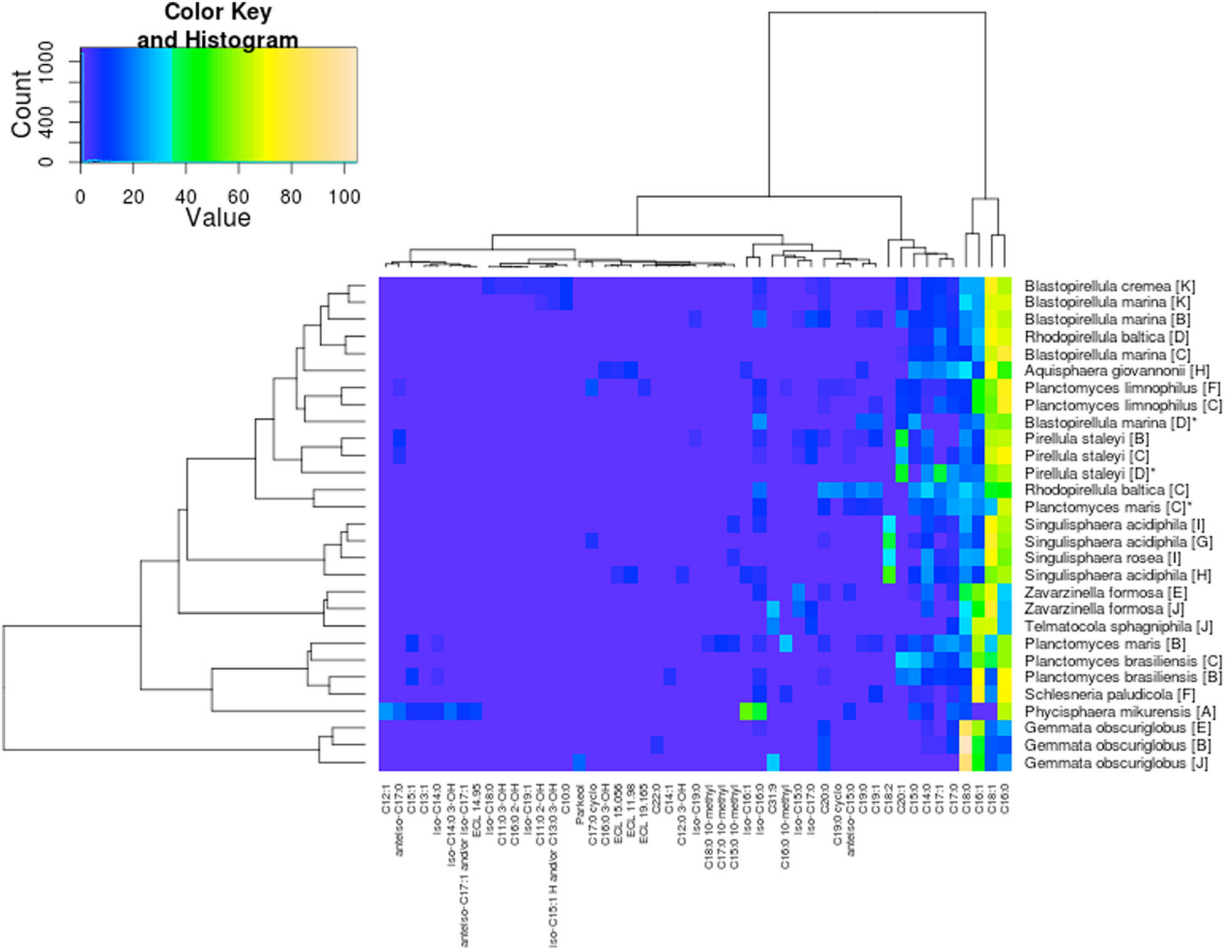
**Heat map of fatty-acid measurements (A**[63]**, B **[65]**, C **[36]**, D **[66]**, E **[68]**, F **[55]**, G **[67]**, H **[70]**, I **[71]**, J **[48]**]) from the analyzed *Planctomycetaceae *(see above).** The heat map was calculated with the R package opm [94] with arcsine-square root transformation and Ward clustering applied. Asterisks denote fatty-acid profiles whose sum deviates more than five percent from 100%.

Kulichevskaya *et al*. [55] used fatty acids as one of the major characteristics that distinguish *Schlesneria* from the genus *Planctomyces* and thus included them in the genus description of *Schlesneria*. To assess whether there is any published evidence for distinguishing *Planctomyces* and *Schlesneria* on the basis of fatty acids as well as to study the reproducibility of the results, the fatty-acid measurements for *Planctomycetes* were comprehensively collected from the literature and re-evaluated (see Figure 6). Caution should however be exercised in the interpretation of the results since different methods have been used in different publications.

The measurements from allegedly the same strains did not always cluster together. *Blastopirellula marina*, *P. maris* and *Rhodopirellula baltica* showed distinct groupings of their respective measurements. The sum of the *B. marina* measurements from Schlesner *et al*. [66] is substantially below 100%, whereas the measurements of *P. maris* from Kerger *et al*. [36] sum up to much less than 100%, indicating missing or overstated values (Figure 6, all strong deviations of the sum from 100% denoted with asterisks). Omitting the questionable data in this respect did not alter the relative positioning of the other measurements (data not shown). The differences between the *R. baltica* measurements from two studies [36,66] might be caused by the distinct growth conditions (temperature and medium). Except for these three species, the fatty-acid analyses of the *Planctomycetes* type strains appeared fairly reproducible (Figure 6).

The analysis of the fatty-acid measurements shows a group comprising *P. brasiliensis*, *P. maris* and *S. paludicola* (disregarding the incomplete *P. maris* data from [66]), whereas *P. limnophilus* does not cluster together with the other *Planctomyces* species. The analysis supports the earlier finding [55] that *S. paludicola* can be differentiated from *P. limnophilus* on the basis of the fatty acids. However, the analysis also shows that *S. paludicola* cannot be differentiated from *P. brasiliensis* and *P. maris*, whose fatty acids were not analyzed in [55]. Rather, the fatty-acid profile of *P. limnophilus* differs from that of the other *Planctomyces* species (and *Schlesneria*), hence the overall similarity in fatty-acid profiles does not entirely reflect the relationships inferred from 16S rRNA gene (Figure 1) and phylogenomic analyses (Figure 4) as well as the GGDC results (Table 6). Thus the evidence for distinguishing *Planctomyces* and *Schlesneria* on the basis of fatty acids given by Kulichevskaya *et al*. [55] cannot be confirmed once all three *Planctomyces* species with cultivated type strains are considered, since the published fatty-acid profile of *S. paludicola* cannot be differentiated from that of *P. brasiliensis* and *P. maris*.

### Taxonomy of the *Planctomyces*-*Schlesneria* complex

Given the positioning of the *Planctomyces* and *Schlesneria* species in the phylogenetic trees (Figure 1, Figure 4), several taxonomic changes could be conducted to render all genera monophyletic. The taxonomy of the group is apparently hampered by the unavailability of a culture of the type species, *P. bekefii*, which was described 90 years ago and based on a few morphological characters [4]. In our view, the scarcity of published information on this species does not allow prediction of its phylogenetic position relative to the other species if a 16S rRNA gene sequence of *P. bekefii* could be obtained (Figure 1). For this reason, the only safeguard against the possibility that phylogenetic analysis of *P. bekefii* would keep *Planctomyces* monophyletic is to place all *Planctomyces* species with an already known 16S rRNA gene (or even genome) sequence into a genus of their own. Indeed, arguing against the removal of other *Planctomyces* species from the genus because the comparison with the type species using contemporary taxonomic methods is impossible would be illogical, because none of the other *Planctomycetes* genera with validly published names have been compared with *P. bekefii* using modern methods either. For instance, there is insufficient evidence against a sister-group relationship between *P. bekefii* and *S. paludicola*, which was described mainly based on the comparison with *P. limnophilus*[55].

Whether or not the three cultivated *Planctomyces* species and *Schlesneria* should be placed in one, two, three or four genera should also be assessed by comparing the divergence among the resulting taxa with those of others. For instance, it would be inconsistent to place all four species, or only two of them, in a single genus if less divergent groups within *Planctomycetes* existed that were nevertheless split into several genera. Divergence was measured directly from the phylogenetic trees (Figure 1, Figure 4) as the maximum subtree height (MaSH) of each subtree without its stem branch. This measure has the advantage that it is on the one hand equivalent to half the maximum pairwise (patristic) distance between organisms placed in a subtree of an ultrametric tree but on the other hand can also be applied if a phylogenetic tree is non-ultrametric, which is much more frequent (Figure 1, Figure 4). Indeed, pairwise distances can be taxonomically misleading because under non-ultrametric conditions less distant organisms need not be more closely related [38,59,69]. Using MaSH, the reference to subtrees guarantees by definition that only monophyletic groups are measured.

The results are shown in Table 7. In the 16S rRNA gene tree, the *P. brasiliensis/maris* and *P. limnophilus/S. paludicola* subtrees were among those subtrees with the smallest MaSH values, but these are more than twice as large as those for the two other single-genus subtrees (containing *Blastopirellula* and *Singulisphaera*, respectively). Further, the subtree containing *Schlesneria* and all *Planctomyces* species yielded a larger MaSH than a number of subtrees that contain up to three genera. In the ML trees inferred from genome-scale data, the *Blastopirellula/Pirellula* and the *Gemmata/Zavarzinella* subtrees showed in same cases larger MaSH values than the *P. brasiliensis/maris* and *P. limnophilus/S. paludicola* subtrees, but in other cases smaller MaSH values. These results indicate that there is no substantial difference between the divergence of the *P. brasiliensis/maris* group, the *P. limnophilus/ S. paludicola* group and *Planctomycetes* groups that comprise two genera. This argues against placing *P. brasiliensis* and *P. maris* in the same genus and against placing *P. limnophilus/S. paludicola* in the same genus, let alone placing all four species in a single genus.

**Table 7:** MaSH values calculated for the ML trees inferred in this study

**Subtree**	**16S rRNA gene**	**Full supermatrix**	**MARE supermatrix**	**Core-gene supermatrix**	**Gene content**	**Ortholog content**
*S. acidiphila*, S. rosea*	0.027	~	~	~	~	~
B. cremea*, *B. marina*	0.032	~	~	~	~	~
*P. brasiliensis*, *P. maris*	0.072	0.414	0.272	0.287	0.166	0.185
*P. limnophilus*, Sc.	0.074	0.382	0.240	0.245	0.193	0.215
*Te*.*, *Za*.	0.086	~	~	~	~	~
*Aq*.*, *Is*.	0.090	~	~	~	~	~
*Aq*.*, *Is*., *S. acidiphila*, S. rosea*	0.100	0.422	0.305	0.309	0.177	0.209
B. cremea*, *B. marina*, *Pi*.	0.101	0.378	0.272	0.267	0.164	0.171
*Ge*., *Te*.*, *Za*.	0.119	0.346	0.280	0.275	0.213	0.236
*Pl*., *Sc*.	0.121	0.480	0.338	0.333	0.198	0.220
B. cremea*, *B. marina*, *Pi*., *Rh*.	0.125	0.539	0.353	0.382	0.379	0.347
*Aq*.*, *Ge*., *Is*., *S. acidiphila*, S. rosea*, *Te*.*, *Za*.	0.245	0.633	0.579	0.522	0.381	0.448
*Aq*.*, *Ge*., *Is*., *Pl*., *Sc*., *S*. *acidiphila*, S. rosea*, *Te*.*, *Za*.	0.297	0.658	0.523	0.522	0.410	0.382
*Aq*.*, *B*. cremea*, *B*. *marina*, *Ge*., *Is*., *Pi*., *Pl*., *Rh*., *Sc*., *S*. *acidiphila*, *S*. rosea*, *Te*.*, *Za*.	0.308	0.734	0.682	0.588	0.427	0.495
*Aq*.*, B. cremea*, *B. marina*, *Ge*., *Is*., *Ph*., *Pi*., *Pl*., *Rh*., *Sc*., *S*. *acidiphila*, S. rosea*, *Te*.*, *Za*.	0.639	1.258	1.118	1.082	0.558	0.649

*Indicate organisms that were only present in the 16S rRNA gene tree.

Phenotypic differences between *P. brasiliensis* and *P. maris* on the one hand and *P. limnophilus* on the other hand also exist; for instance, the fatty-acid profiles of the former differ from that of the latter in the same way (Figure 6) as the *S. paludicola* profile differs from the one of *P. limnophilus*[55]. *S. paludicola* differs from all three *Planctomyces* species with respect to the number of flagella of the daughter cells [55]. Moreover, *P. brasiliensis* and *P. maris* contain *sym*-homospermidine as polyamine, whereas *P. limnophilus* contains putrescine instead (Table 8). *P. maris* differs from *P. brasiliensis* because only the latter contains spermidine (Table 8). Given that four *Planctomycetes* placed in distinct genera (*Blastopirellula*, *Gemmata*, *Isosphaera*, *P. maris*) possess the same polyamine pattern (Table 8) the differences between the *Planctomyces* species appear significant. Regarding polar lipids, *P. maris* contains phosphatidyl-monomethylethanolamine and phosphatidyl-dimethylethanolamine, which are not produced by *P. brasiliensis* (Table 8). Compared to *Aquisphaera* and *Gemmata*, between which no differences regarding their polar-lipid pattern have been reported [69,65], but which are placed in distinct genera, the differences between the *Planctomyces* species again appear significant.

**Table 8:** **Polyamines**[96 ]** and polar lipids **[48,65,70,95]** for several *Planctomycetaceae *** **as reported in the literature**

	**Polyamines**				**Polar lipids**^**1**^					
	**Cadaverine**	**Putrescine**	**Spermidine**	***sym*-Homospermidine**	**DPG**	**Glyl**	**PC**	**DPE**	**PG**	**MPE**
*A. giovannonii*[70]	NR^2^	NR	NR	NR	+	-	+	-	+	-
*B. cremea*[95]	NR	NR	NR	NR	-	-	-	-	+	-
*B. marina*[40,65,66]	-	-	-	+	+	-	-	-	+	-
*G. obscuriglobus*[40,65]	-	-	-	+	+	-	+	-	+	-
*I. pallida*[40]	-	-	-	+	NR	NR	NR	NR	NR	NR
*P. staleyi*[40,65,66]	-	-	+	+	+	-	-	-	+	+
*P. brasiliensis*[40,65]	-	-	+	+	+	+	-	-	+	-
*P. limnophilus*[40,65]	-	+	+	-	+	+	-	-	+	-
*P. maris*[40,65]	-	-	-	+	+	+	+	+	+	+
*R. baltica*[40,65,66]	+	+	-	+	+	+	+	+	+	-
*S. acidiphila*[48]	NR	NR	NR	NR	-	-	+	-	+	-
*S. rosea*[48]	NR	NR	NR	NR	-	-	+	-	+	-

^1^Phospholipids: DPG Diphosphatidylglygerol; Glyl Glycolipid; PC Phosphatidylcholine; DPE Phosphatidyl-dimethylethanolamine; PG Phosphatidylglycerol; MPE Phosphatidyl-monomethylethanolamine.

^2^Not reported in the literature.

We conclude that both genomic and phenotypic analyses according to both qualitative and quantitative criteria support the placement of the three *Planctomyces* species with cultivated type strains into genera separate from each other and from *Schlesneria*.

### Taxonomic consequences

As explained in detail above, the differences in the reported G + C contents of *G. obscuriglobus, S. acidiphila* and *Z. formosa* from the ones calculated from their genome sequences justify an emendation of their species descriptions and in the case of *Gemmata* an emendation of the genus description (which referred to the G + C content).

Moreover, the range of G + C content values determined for the genera now placed within *Planctomycetaceae* (*Planctomycetales*) is in conflict with the description of the family (and order) as given by Schlesner and Stackebrandt 1986 [27]. In addition, an optimal growth temperature of 41°C is reported for *Isosphaera*[55], which is considerably higher than the one reported in the description of the family (and order). For *Schlesneria*, *Singulisphaera*, *Telmatocola* and *Zavarzinella* a slightly lower optimal growth temperature is reported, i.e. 20°C upward [48,55,67,68,71]. Furthermore, for the genera *Schlesneria* and *Zavarzinella* it was reported that the daughter cells are motile by means of two [55] or one to two flagella [68], respectively, whereas the description of the family (and order) only considered the presence of one flagellum.

Whereas these discrepancies call for an emendation of the family *Planctomycetaceae*, phylogenomic (Figure 4) and 16S rRNA gene analyses (Figure 1) show a monophyletic group comprising the genera with high G + C content. The remaining genera appear unresolved in the 16S rRNA gene tree (Figure 1) but as a maximally supported clade comprising the genera *Planctomyces*, *Schlesneria*, *Blastopirellula*, *Pirellula* and *Rhodopirellula* in the phylogenomic tree (Figure 4). This would support a splitting of the quite heterogeneous *Planctomycetaceae* into two families, but the unknown position of the type species, *P. bekefii,* of the type genus means that a more narrowly defined *Planctomycetaceae* may not include *P. bekefii*. For this reason, we refrain from generating a novel family to accommodate the high-G + C genera.

### Emended description of the genus *Gemmata* Franzmann and Skerman (1985)

The description is the one given by Franzmann and Skerman [61] with the following modification.

The G + C content is about 67%.

### Emended description of the species *Gemmata obscuriglobus* Franzmann and Skerman (1985)

The description of the species *Gemmata obscuriglobus* is the one given by Franzmann and Skerman [61], with the following modification.

The G + C content is 67%.

### Emended description of the species *Singulisphaera acidiphila* Kulichevskaya *et al*. (2008)

The description of the species *Singulisphaera acidiphila* is the one given by Kulichevskaya et al. (2008) [67], with the following modification.

The G + C content is 62%.

### Emended description of the species *Zavarzinella formosa* Kulichevskaya *et al*. 2009

The description of the species *Zavarzinella formosa* is the one given by Kulichevskaya et al. (2009) [68], with the following modification.

The G + C content is 59%.

### Emended description of the order *Planctomycetales* Schlesner and Stackebrandt 1986

The description of the order *Planctomycetales* is the one given by Schlesner and Stackebrandt [27], with the following modification.

Daughter cells may be motile by means of one or two flagella. The G + C content ranges between about 50% and about 70%. The growth temperature optimum ranges between 20°C and 40°C.

### Emended description of the family *Planctomycetaceae* Schlesner and Stackebrandt 1986

The description of the family *Planctomycetaceae* is the one given by Schlesner and Stackebrandt [27], with the following modification.

Daughter cells may be motile by means of one or two flagella. The G + C content ranges between about 50% and about 70%. The growth temperature optimum ranges approximately between 20°C and 40°C.

### Description of *Rubinisphaera* gen. nov

*Rubinisphaera* (*Ru.bi.ni.sphaer’a* N. L. fem. n. *Rubinia* named in honor of Edward Rubin, an American geneticist and medical researcher, who played a key role in initiating the Genomic Encyclopedia of *Archaea* and *Bacteria* project at the DOE Joint Genome Institute (JGI)*;* NL fem. n. *sphaerae*, a ball, globe, sphere; N. L. fem. n. *Rubinisphaera*, a spherical organism named after Edward Rubin).

The features are the ones of the type species. Characteristic polyamines are *sym*-homospermidine and spermidine. The polar lipids phosphatidyl-monomethylethanolamine and phosphatidyl-dimethylethanolamine are absent. Major fatty acids are C_16:0_ and C_16:1_ ω7c. The G + C content is about 56%.

The type species is *Rubinisphaera brasiliensis. Rubinisphaera* belongs to *Planctomycetaceae*.

### Description of *Rubinisphaera brasiliensis* (Schlesner 1990) comb. nov.

*Rubinisphaera brasiliensis* (*bra.si.lien’sis* L. m. adj. pertaining to the country of Brazil).

Basonym: *Planctomyces brasiliensis* Schlesner 1990.

The genus *Rubinispharea* is comprised of one species, *Rubinisphaera brasiliensis*. The characteristics of the species are given in the genus description and the description given by Schlesner (1989) [1].

The type strain is IFAM 1448^T^ (=ATCC 49424 = DSM 5305 = JCM 21570 = NBRC 103401).

### Description of *Planctopirus* gen. nov.

*Planctopirus* (*Planc.to.pi’rus* Gr. adj. *planktos*, wandering, floating; L. maS. n. *pirus*, a pear tree, referring to the shape of the cells, which also occur in bundles; NL fem. n. *Planctopirus*, floating pear tree).

The features are the ones of the type species. Characteristic polyamines are putrescine and spermidine. Major fatty acids are C_16:0_, C_16:1_ ω7c and C_18:1_ ω9c. The G + C content is about 54%.

The type species is *Planctopirus limnophilus. Planctopirus* belongs to *Planctomycetaceae*.

### Description of *Planctopirus limnophilus* (Hirsch and Müller 1986) comb. nov.

*Planctopirus limnophilus* (*lim.no’phi.la* Gr. n. *limnos* lake; Gr. adj. *philus* loving; NL adj. *limnophila* lake loving).

Basonym: *Planctomyces limnophilus* Hirsch and Müller 1986.

The genus *Planctopirus* is comprised of one species, *Planctopirus limnophilus*. The characteristics of the species are given in the genus description and the description given by Hirsch and Müller (1986) [2].

The type strain is Mü 290^T^ (=ATCC 43296 = DSM 3776 = IFAM 1008).

### Description of *Gimesia* gen. nov.

*Gimesia* (*Gi.me’si.a* N. L. fem. n. *Gimesia* named in honor of Nándor István Gimesi, a Hungarian plant physiologist and plant morphologist, who first reported organisms now placed in the phylum *Planctomycetes*).

The features are the ones of the type species. The only major polyamine component is *sym*-homospermidine. The polar lipids contain phosphatidyl-monomethylethanolamine and phosphatidyl-dimethylethanolamine. Major fatty acids are C_16:0_ and C_16:1_ ω7c. The G + C content is about 50%.

The type species is *Gimesia maris. Gimesia* belongs to *Planctomycetaceae*.

### Description of *Gimesia maris* (ex Bauld and Staley 1976) comb. nov.

*Gimesia maris* (*mar’is* L. gen. noun *maris* of the sea).

Basonym: *Planctomyces maris* (ex Bauld and Staley 1976) Bauld and Staley 1980.

The genus *Gimesia* is comprised of one species *Gimesia maris*. The characteristics of the species are given in the genus description and the description given by Bauld and Staley (1976) [97] and Bauld and Staley (1980) [3].

The type strain is 534-30^T^ (=ATCC 29201 = DSM 8797).

## Conclusion

This study presents the genome sequence for the *P. brasiliensis* type strain IFAM 1448T, whose physiological and genomic features are reviewed in detail. Results from phylogenomic analyses including all available *Planctomycetaceae* genomes disagree with the present circumscription of the genera *Planctomyces* and *Schlesneria*. The revisited 16S rRNA gene and phenotypic data from the literature neither support the current classification. A quantitative comparison of phylogenetic and phenotypic aspects suggest the formation of three new genera (for which we propose the names *Gimesia*, *Planctopirus* and *Rubinisphaera*) to accommodate *P. maris*, *P. limnophilus* and *P. brasiliensis*, respectively. Considerable differences between the reported G + C content of *Gemmata obscuriglobus*, *Singulisphaera acidiphila* and *Zavarzinella formosa* and G + C content calculated from their genome sequences are found, suggesting the emendation of their species descriptions. The range of G + C values reported for the *Planctomycetaceae* (*Planctomycetales*) indicate that the descriptions of the family and the order should be emended.

## Competing interests

The authors declare that they have no competing interests.

## Author's contributions

CS, BA and MG conducted the phylogenomic studies. CS, BJT, RM, BA, PH, MG and HPK drafted the manuscript. MR performed the laboratory experiments. ML, MN, AL, JFC, LG, SP, MH, KL, IP, KM, NI, AP, AC, KP, CDJ, LH, ML, JCD, TW, JAE, VM and NCK sequenced, assembled and annotated the genome. All authors read and approved the final manuscript.
